# A Genetic Study of Cerebral Atherosclerosis Reveals Novel Associations with *NTNG1* and CNOT3

**DOI:** 10.3390/genes12060815

**Published:** 2021-05-26

**Authors:** Selina M. Vattathil, Yue Liu, Nadia V. Harerimana, Adriana Lori, Ekaterina S. Gerasimov, Thomas G. Beach, Eric M. Reiman, Philip L. De Jager, Julie A. Schneider, David A. Bennett, Nicholas T. Seyfried, Allan I. Levey, Aliza P. Wingo, Thomas S. Wingo

**Affiliations:** 1 Department of Neurology, Emory University School of Medicine, Atlanta, GA 30322, USA; selina.maria.vattathil@emory.edu (S.M.V.); yue.liu2@emory.edu (Y.L.); nadia.victoria.harerimana@alumni.emory.edu (N.V.H.); ekaterina.sergeevna.gerasimov@emory.edu (E.S.G.); alevey@emory.edu (A.I.L.); 2 Department of Psychiatry, Emory University School of Medicine, Atlanta, GA 30322, USA; alori@emory.edu; 3 Department of Pathology, Banner Sun Health Research Institute, Sun City, AZ 85351, USA; thomas.beach@bannerhealth.com; 4 Banner Alzheimer’s Institute, Arizona State University and University of Arizona, Phoenix, AZ 85351, USA; eric.reiman@bannerhealth.com; 5 Center for Translational and Computational Neuroimmunology, Department of Psychiatry, Columbia University Medical Center, New York, NY 10032, USA; pld2115@cumc.columbia.edu; 6 Rush Alzheimer’s Disease Center, Rush University Medical Center, Chicago, IL 60612, USA; Julie_A_Schneider@rush.edu (J.A.S.); David_A_Bennett@rush.edu (D.A.B.); 7 Department of Biochemistry, Emory University School of Medicine, Atlanta, GA 30329, USA; nseyfri@emory.edu; 8 Division of Mental Health, Atlanta VA Medical Center, Decatur, GA 30033, USA; 9 Department of Human Genetics, Emory University School of Medicine, Atlanta, GA 30322, USA

**Keywords:** cerebral atherosclerosis, genome-wide association, mediation

## Abstract

Cerebral atherosclerosis is a leading cause of stroke and an important contributor to dementia. Yet little is known about its genetic basis. To examine the association of common single nucleotide polymorphisms with cerebral atherosclerosis severity, we conducted a genomewide association study (GWAS) using data collected as part of two community-based cohort studies in the United States, the Religious Orders Study (ROS) and Rush Memory and Aging Project (MAP). Both studies enroll older individuals and exclude participants with signs of dementia at baseline. From our analysis of 1325 participants of European ancestry who had genotype and neuropathologically assessed cerebral atherosclerosis measures available, we found a novel locus for cerebral atherosclerosis in *NTNG1*. The locus comprises eight SNPs, including two independent significant SNPs: rs6664221 (*β* = −0.27, 95% CI = (−0.35, −0.19), *p* = 1.29 × 10^−10^) and rs10881463 (*β* = −0.20, 95% CI = (−0.27, −0.13), *p* = 3.40 × 10^−8^). We further found that the SNPs may influence cerebral atherosclerosis by regulating brain protein expression of CNOT3. CNOT3 is a subunit of CCR4−NOT, which has been shown to be a master regulator of mRNA stability and translation and an important complex for cholesterol homeostasis. In summary, we identify a novel genetic locus for cerebral atherosclerosis and a potential mechanism linking this variation to cerebral atherosclerosis progression. These findings offer insights into the genetic effects on cerebral atherosclerosis.

## 1. Introduction

Cerebral atherosclerosis is a leading cause of cerebral infarction and hemorrhage. Like other types of atherosclerosis, it is recognized as a multifactorial disease with risk determined by inherited genetic variation, environmental factors, and interactions among these features [1]. Major modifiable risk factors for atherosclerosis in general include dyslipidemia, hypertension, obesity, diabetes, and cigarette smoking, but the relative contribution of each risk factor varies for different subtypes of atherosclerosis [2]. Cerebral atherosclerosis risk varies across ancestry groups, with especially high risk observed in populations of East Asian, African, and Hispanic ancestry [3,4,5,6]. While much of the variation in risk is attributable to differences in healthcare, diet, and other environmental factors, the SNP-based heritability ($h_{SNP}^{2}$) for intracranial carotid artery calcification, a common manifestation of cerebral atherosclerosis, has been estimated at 0.47 [7], underscoring the contribution of common genetic variation to cerebral atherosclerosis risk. While genetic variation has been investigated in a small number of studies targeting single nucleotide polymorphisms (SNPs) and indels in candidate genes [8,9,10,11], these variants do not fully explain the genetic component of cerebral atherosclerosis risk. We recently conducted a proteome-wide association study (PWAS) and protein co-expression network analysis of cerebral atherosclerosis in which we found evidence that cerebral atherosclerosis was associated with increased oligodendrocyte differentiation and shared differentially expressed protein modules with Alzheimer’s disease (AD) independent of AD’s hallmark pathologies, neuritic plaques, and neurofibrillary tangles [12]. Here, we set out to identify genetic variants associated with cerebral atherosclerosis by conducting a genome-wide association study (GWAS) of 1325 people collected in two longitudinal studies of aging and cognitive decline. We identified a novel genetic locus for cerebral atherosclerosis and found evidence that it may act by regulating brain protein levels of CNOT3. The CNOT3 protein is a subunit of CCR4−NOT, a master regulator of mRNA stability and translation and an important complex for cholesterol homeostasis. We tested the association in two independent datasets and found that the locus showed consistent association in a community-based cohort but not in a clinical referral cohort. Follow-up population-based studies and functional studies will improve our understanding of the role of this genetic locus in cerebral atherosclerosis. 

## 2. Materials and Methods

### 2.1. Discovery Cohort

Participants were recruited by the Religious Orders Study (ROS) and Rush Memory and Aging Project (MAP) [13] (Table 1), together referred to as ROS/MAP. Both studies are longitudinal clinical-pathologic community-based cohort studies of cognitive decline, dementia, and aging and administer detailed cognitive and clinical evaluations to participants on an annual basis. ROS enrolls priests, nuns, and monks aged greater than 53 years at locations throughout the United States, while MAP recruits lay people from assisted living facilities in the greater Chicago area. Both studies exclude individuals with signs of dementia at baseline evaluation. All participants are organ donors, provided informed consent, and signed an Anatomical Gift Act and a repository consent to allow their data and biospecimens to be repurposed. An Institutional Review Board of Rush University Medical Center approved the studies. The ROS/MAP studies are ongoing; this analysis included 1325 participants of European ancestry who had cerebral atherosclerosis measures and sequence data available at the time of analysis.

### 2.2. Cerebral Atherosclerosis Assessment

Cerebral atherosclerosis was pathologically assessed by visual inspection of the vessels in the circle of Willis including vertebral, basilar, posterior cerebral, middle cerebral, and anterior cerebral arteries and their proximal branches [14]. Cerebral atherosclerosis severity was scored on a scale of 0 to 3 based on the number of arteries involved and the extent of involvement of each artery. A score of zero indicates no significant atherosclerosis was observed. A score of 1 (mild) indicates small amounts of luminal narrowing in up to several arteries without significant occlusion. A score of 2 (moderate) indicates luminal narrowing in up to half of all visualized major arteries with less than 50% occlusion of any single vessel. Lastly, a score of 3 (severe) indicates luminal narrowing in more than half of all visualized arteries and/or more than 75% occlusion of one or more vessels [14].

### 2.3. Genotyping and SNP Association Testing

Samples were genotyped using either the Affymetrix GeneChip 6.0 (Santa Clara, CA, USA) (~900,000 SNPs, *n* = 1132) or the Illumina HumanOmniExpress (San Diego, CA, USA) (~700,000 SNPs, *n* = 193) as described previously [15]. Genotypes were then imputed to the 1000 Genomes Project Phase 3 using the Michigan Imputation Server [16]. Genetic principal components (PCs) were calculated using EIGENSTRAT [17] to characterize population structure and to select participants of European ancestry. One sample was an outlier (> 6 standard deviations from the top 10 PCs) and was excluded from analysis. Kinship analysis using KING [18] confirmed all subjects were unrelated (kinship coefficient <1/32). SNPs that failed to meet the following criteria were excluded: genotype missingness <5%, minor allele frequency (MAF) >5%, Hardy-Weinberg equilibrium *p*-value >10^−5^, and genotype imputation *r*^2^ > 0.3. After imputation and participant and SNP filtering, a total of 1325 samples and 8,119,107 SNPs were available for analysis.

Each SNP was tested for association with cerebral atherosclerosis by linear regression analysis using the implementation in PLINK [19]. The model treated cerebral atherosclerosis score as a semiquantitative outcome variable, treated allele effects as additive, and included the following covariates: sex, age at death, genotyping array, and 10 genetic PCs. To calculate the genomic inflation factor λ, we converted the *p*-values to chi-square statistics assuming 1 degree of freedom, then calculated the genomic inflation factor as the ratio of the observed median chi-square statistic to the median value expected under the null hypothesis. We annotated SNPs and identified independent significant associations using FUMA [20] v1.3.5e using the conventional genomewide significance threshold for association test *p*-value (5 × 10^−8^) [21] and *r*^2^ value of 0.6, and using 1000G Phase 3 EUR as the reference population. Regional plots were generated using LocusZoom [22] v1.4. Power analysis was conducted using code from https://github.com/kaustubhad/gwas-power, accessed on 22 April 2020, commit e0dddff, based on formulae presented in Appendix A of [23].

### 2.4. Meta-Analysis

We meta-analyzed the ROS/MAP results with results from two independent datasets. The first independent dataset included participants of the Arizona Study of Aging and Neurodegenerative Disorders, a longitudinal clinical-pathologic study of normal aging, Alzheimer’s disease (AD), and Parkinson’s disease (PD) run by the Banner Sun Health Research Institute [24]. The study population consists of cognitively unimpaired volunteers from retirement communities in northwest greater Phoenix, Arizona with some directed recruitment of participants with AD and PD through neurologists in the metropolitan Phoenix and Tucson areas [24]. Over 90% of participants are of European ancestry. All subjects or their legal representatives sign a Banner Sun Health Research Institute Institutional Review Board-approved informed consent form allowing both clinical assessments during life, several options for brain and/or bodily organ donation after death, and usage of donated biospecimens for approved future research. Circle of Willis atherosclerosis was assessed as the extent of atherosclerotic plaque visible on gross external examination of the circle of Willis. A score of none, mild, moderate, or severe was assigned according to a schematic template [25]. DNA from post-mortem brain tissue was extracted using Qiagen GenePure kit and genotyped using the Affymetrix Precision Medicine Array following the manufacturer’s protocol. Genotypes were imputed and all participants were verified to be of European ancestry and unrelated as described above for the discovery dataset. The SNPs were filtered using the same criteria described above for the discovery dataset. A total of 154 participants with cerebral atherosclerosis assessment and genotype data were available for this analysis.

The second independent dataset included individuals from 31 Alzheimer’s Disease Centers (ADCs) with phenotypes available from the National Alzheimer’s Coordinating Center (NACC) database [26] and genotyping data generated by the Alzheimer’s Genetics Consortium (ADGC) [27]. Each individual ADC received informed consent from their participants and approval from their institutional review board. Atherosclerosis of the circle of Willis was scored as none, mild, moderate, or severe. All ADCs used the same forms for scoring. The autopsies were conducted between 2005 and 2020, and genotyping was performed in 7 batches. A total of 1914 participants with cerebral atherosclerosis assessment and genotype data were available for this analysis.

We calculated association test statistics in these two datasets using the same framework applied to ROS/MAP, with the following covariates: sex, age at death, and 4 genetic PCs (because only the first 4 PCs were significant) for the Banner dataset; and sex, age at death, 10 genetic PCs, and genotyping batch for the ADGC dataset. We then performed inverse variance weighted meta-analysis using METAL [28].

### 2.5. Protein-Protein Interaction

Lists of pairwise protein interactions were downloaded from the BioGRID database (v3.5.179, 29 October 2019) [29] and filtered for interactions containing only human gene symbols of interest.

## 3. Results

Table 1 provides demographic data for the 1325 participants included in the discovery analysis. Mean age at death was 89 years. Two thirds of the participants were females, and all were of European ancestry. Almost half of participants were diagnosed with mild cerebral atherosclerosis, while 17% had no or slight cerebral atherosclerosis and 28% had moderate atherosclerosis. Another 7% of participants had severe atherosclerosis.

**Figure 1:**
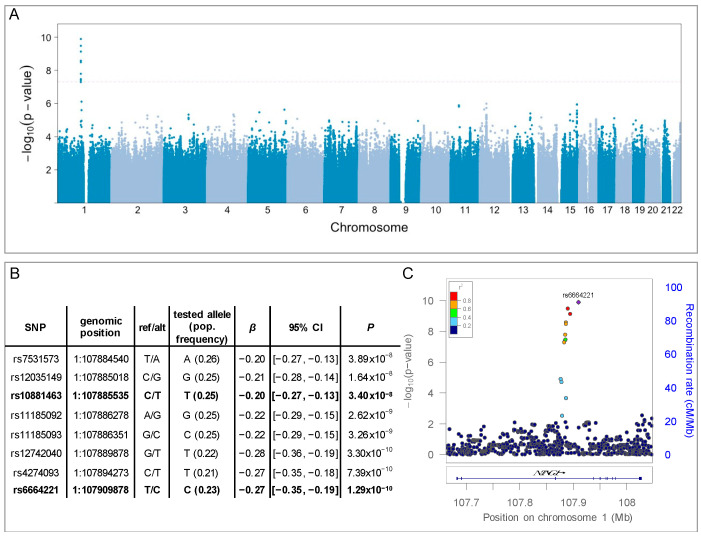
GWAS results. (**A**) Manhattan plot of *p*-values per SNP plotted by genomic position. Dotted line marks genome-wide significance threshold (*p* < 5 × 10^−8^). A locus on chromosome 1 reached genome-wide significance. (**B**) Genomic locations, allele information, and *β* estimates for the eight significant SNPs. The reported population allele frequencies were estimated from the 1000G CEU samples. For all eight SNPs, the minor allele was the tested allele. Independent SNPs are highlighted in bold. (**C**) Detail of significant locus and surrounding genomic region, including intron-exon diagram for *NTNG1*. The diamond indicates the top SNP, and color of round points indicates the level of linkage disequilibrium (measured as *r*^2^) with the top SNP, estimated from the 1000G EUR reference population.

**Figure A1:**
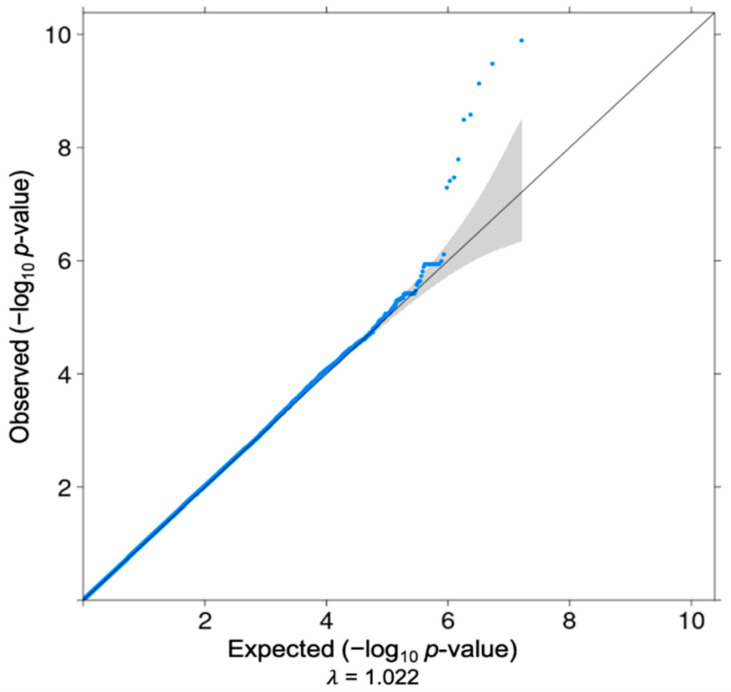
Quantile-Quantile plot of observed *p*-value distribution. Gray shading shows 95% confidence intervals for 1000 most significant *p*-values expected under the null hypothesis.

**Figure A2:**
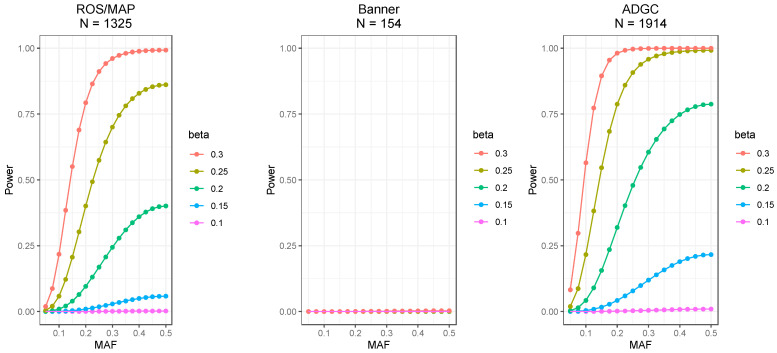
Power analysis. Plots shows power at *p*-value < 5 × 10^−8^ for a range of effect size (β) values across the full range of MAF values. Overall, the results for the ROS/MAP discovery analysis suggest above 50% power to detect alleles with effects size of 0.25 down to ~25% MAF (panel A). The effect size of significant SNPs in ROS/MAP ranged from *β* values of −0.27 to −0.20 for alleles with approximately 20% MAF; thus, panel B suggests essentially no power to detect those SNPs in the Banner dataset whereas panel C suggests ~32–90% power to detect those SNPs in ADGC.

To understand whether cerebral atherosclerosis has an inherited risk, we performed a GWAS of cerebral atherosclerosis using the pathologic outcome measured in ROS/MAP (Table 1). Power analysis showed >50% power to detect SNP associations at *p*-value < 5 × 10^−8^ for a SNP with MAF of ~0.25 and effect size of 0.25 (Appendix A Figure A2). The quantile-quantile (QQ) plot of the observed *p*-values (Appendix A Figure A1) showed no evidence of overall inflation of the association test statistics (genomic inflation factor λ = 1.02). Eight single-nucleotide polymorphisms (SNPs) were associated with cerebral atherosclerosis at a genome-wide significant level after adjusting for sex, age, genotyping array, and 10 genetic PCs (*p* < 5 × 10^−8^, N = 1325) (Figure 1A). These 8 SNPs are located on chromosome 1 in the gene *NTNG1*, which encodes a member of the netrin family of axon guidance proteins that is involved in synapse formation and neurite guidance [30]. While the eight SNPs are in moderate to high LD with each other, using the annotation tool FUMA [20], we identified two independent SNPs at *r*^2^ < 0.6 (Figure 1B): rs6664221 (*β* = −0.27, *p* = 1.29 × 10^−10^; N = 1325) and rs10881463 (*β* = −0.20, *p* = 3.40 × 10^−8^, N = 1325). For both SNPs, the minor allele was associated with less severe cerebral atherosclerosis (Figure 1C).

We tested sites that were genome-wide significant in two independent datasets. The first dataset comprised 154 participants with post-mortem gross visual assessment of circle of Willis atherosclerosis from individuals of European descent recruited by Banner Sun Health Research Institute [24] (henceforth referred to as the Banner cohort). The median age-at-death of these participants was 86.0 years. Although none of the variants reached significance in this dataset, all eight variants showed a consistent direction of effect and after meta-analyzing the cohorts, we observed a more significant *p* value for every SNP (Table 2). The second independent dataset comprised 1914 participants from 31 ADCs genotyped by ADGC and with neuropathologic data available from NACC. None of the eight SNPs were significantly associated with cerebral atherosclerosis in this dataset or in a meta-analysis of all three datasets (Appendix A Table A1). We note, however, that the ADCs have different recruitment strategies that resemble clinical referral cohorts compared to ROS/MAP and Banner cohorts, which are considered community-based cohorts. Differences in recruitment and clinical characteristics could explain the lack of signal in the ADGC dataset.

To understand whether differences in recruitment strategies may underlie differences in genetic associations, we compared clinical characteristics for the three datasets (Appendix A, Table A2 and Table A3). There were differences in the prevalence of infarcts (both micro and macroscopic), prevalence of dementia (especially non-Alzheimer’s dementia), and Braak score between the ROS/MAP and ADGC datasets. Similar differences have been observed previously between community-based versus clinic-based neuropathology cohorts [31]. Given the differences in clinical characteristics and recruitment strategies, we opted to focus on the results from the two community-based cohorts (Table 2).

**Figure 2:**
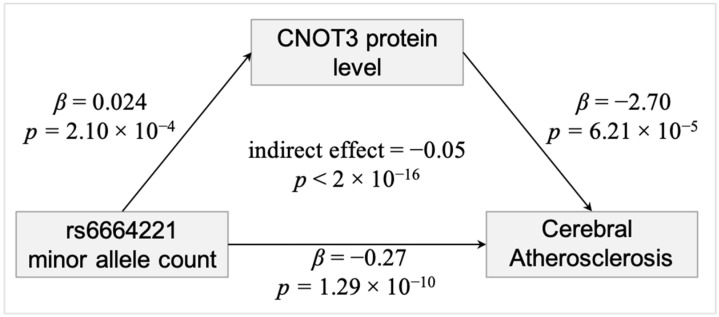
CNOT3 partially mediates the effect of variation at rs6664221 on cerebral atherosclerosis. The effects for rs10881463 are similar.

To explore potential mechanisms for the two lead SNPs that were associated with cerebral atherosclerosis from the meta-analysis of the community-based studies, we tested whether they were associated with RNA and protein expression of *NTNG1*, the gene in which they are located. We found no evidence that they are either eQTL for *NTNG1* mRNA level using 580 individuals from the largest brain eQTL dataset by Sieberts et al. [32], or pQTL for NTNG1 protein using 352 individuals with proteomics measurements in our brain proteomic dataset [33]. Because SNPs can influence gene expression by acting proximally or distally, we then examined whether these two SNPs influence RNA or protein expression of 237 genes that showed differential protein expression in a recent proteome-wide association study (PWAS) of cerebral atherosclerosis [12]. We found that both SNPs are distal pQTL for CNOT3|O75175 (rs10881463: *β* = 0.024, *p* = 3.11 × 10^−5^, Benjamini-Hochsberg (BH) adjusted *p* = 0.0147, N = 352; rs6664221: *β* = 0.024; *p* = 2.10 × 10^−4^; BH adjusted *p* = 0.0497; N = 352). We used formal mediation analysis to test the hypothesis that the SNPs influence cerebral atherosclerosis risk through their effect on CNOT3. The assumptions for mediation analysis were met for both SNPs: they were associated with cerebral atherosclerosis and CNOT3 protein level, and CNOT3 protein level was associated with cerebral atherosclerosis after adjusting for 8 other neuropathologies (Figure 2). The results suggest that the SNPs may influence cerebral atherosclerosis risk partially through CNOT3 (rs6664221: mean indirect effect = −0.05, 95% CI = (−0.0952, −0.01), bootstrap *p* < 0.004; rs10881463: mean indirect effect = −0.05, 95% CI = (−0.0959, −0.02), bootstrap *p* < 2 × 10^−16^; N = 352). Notably, CNOT3 protein has evidence for physical interactions in the BioGRID dataset [29] with two proteins we previously found associated with cerebral atherosclerosis in human brain, HNRNPLL and RTF1 [12].

## 4. Discussion

We performed the first GWAS of pathologically defined cerebral atherosclerosis and identified a novel genetic association with SNPs within an intron of the gene *NTNG1*. *NTNG1* is a member of a family of axon guidance molecules, highly expressed in brain and kidney, and notable for its many isoforms [34]. A few intriguing genetic associations of *NTNG1* appear in the literature. First, we note that other genetic variants in *NTNG1* have been associated with schizophrenia risk in people of Japanese, Han Chinese, and European ancestry [35,36,37,38]. Those variants, however, span differentially spliced exons, while the variants reported here fall between exons common to all isoforms. Second, we note that variation at one of the significant SNPs in this analysis, rs11185092, has been associated with body mass index (BMI), the most common measure of obesity, in three prior GWAS meta-analyses [39,40,41]. The shared genetic association between cerebral atherosclerosis and BMI is intriguing and complex. Obesity and related traits such as diabetes and reduced physical activity are considered risk factors for atherosclerosis. While it is unclear whether BMI provides an independent risk for cerebral atherosclerosis (reviewed here [42]), the lack of a clear association may reflect study design rather than true lack of association [43]. The results of the published BMI meta-analyses indicate the influence of rs11185092 on BMI is subtle, consistent with the current understanding of obesity as a complex polygenic trait. We did not find a significant association between BMI and rs11185092 in our dataset, which is expected given our sample size and the small effect sizes reported in the meta-analyses. Overall, the results suggest that obesity and cerebral atherosclerosis may share biological pathways and genetic underpinnings.

To try to understand the mechanistic relationship between the *NTNG1* locus and cerebral atherosclerosis, we tested the two independent significant SNPs for proximal and distal eQTL and pQTL effects on brain RNA and protein levels of *NTNG1* and 237 other genes that showed differential protein expression in cerebral atherosclerosis in a previous proteomic analysis in human brain [12]. While we found no link between the variants and RNA levels of any genes, we did find that the variants correlated with levels of CNOT3 protein. For both independent significant SNPs, a higher copy number of the minor allele is associated with lower cerebral atherosclerosis severity and a higher CNOT3 level in the brain, and formal mediation analysis suggests that the genetic variants may influence cerebral atherosclerosis by regulating brain protein expression of CNOT3. CNOT3 encodes a subunit of CCR4−NOT, which is a master regulator of mRNA stability and translation [44,45,46] and an important complex for cholesterol homeostasis and steroid hormone synthesis [47]. A study in mice found that Cnot3 haploinsufficiency resulted in higher metabolism, lower serum triglycerides, and obesity resistance in response to a high-fat diet, and preferentially upregulated fat oxidation genes and downregulated lipogenic genes [48]. CNOT3 is also involved in necroptosis and B cell development [49,50].

We acknowledge that our study has both strengths and weaknesses. A major strength of our study is the use of neuropathologically assessed intracranial atherosclerosis. Intracranial and extracranial atherosclerosis are sometimes, but not always, coincident [51]. Thus, while extracranial atherosclerosis is easier to measure, it is not a good proxy for intracranial cerebral atherosclerosis. Additionally, the use of standardized neuropathologic assessment instead of imaging-based phenotyping likely leads to reduced variance and a more precise measurement of cerebral atherosclerosis severity, which likely helps reduce our signal-to-noise-ratio.

A second strength is the sampling of community-based individuals without ascertainment based on a previous stroke. Of the few prior studies of atherosclerosis that focus specifically on intracranial atherosclerosis, most include only subjects who have had a previous stroke. While cerebral atherosclerosis is a strong risk factor for a stroke, it may also exist as an asymptomatic condition. Even among people with intracranial atherosclerosis, stroke risk is modified by features such as severity of stenosis and collateral flow status [51], and therefore, those studies will be biased toward individuals with more severe distributions for those features. Thus, our analyses using a sample from the general population may yield different insights than analyses using stroke patients only. On the other hand, the uniqueness of the dataset means we met a challenge finding appropriate datasets for replication. The *NTNG1* signal in the Banner cohort, which is also mostly a community-based cohort, was consistent with the signal observed in ROS/MAP and therefore supports the association, notwithstanding the modest sample size. However, we did not find evidence for replication in the ADGC dataset. The ROS/MAP studies use a relatively high minimum age of enrollment of 65 years and additionally require that participants show no signs of dementia at enrollment, while individuals enrolled at ADCs typically have a history of dementia at baseline [31]. Given the significant difference in recruitment strategy employed by the community-based ROS/MAP study and the ADCs, the absence of the *NTNG1* signal in ADC participants may reflect differences in underlying features of these datasets. We observe that, compared to ROS/MAP, the ADGC cohort has a lower prevalence of infarcts, higher prevalence of dementia, especially non-Alzheimer’s dementia, and higher mean Braak stage, which suggests there are important differences in clinical characteristics between the cohorts. Nevertheless, we do not know whether these differences were directly responsible for the lack of association or whether our finding in ROS/MAP and Banner datasets was simply a false-positive. As the ROS/MAP studies are ongoing, it may be possible to reassess in this cohort with a larger sample size in the future. Follow-up in additional large community-based cohorts, and especially in datasets including more ancestrally diverse people (since the examined cohorts are overwhelmingly of European ancestry), is crucial to provide independent validation of the current findings, discover additional variants, and define whether variants are shared or specific to populations.

## 5. Conclusions

In conclusion, we identified a novel risk locus in *NTNG1* that may contribute to cerebral atherosclerosis severity by mediating brain protein levels of CNOT3, a sub-unit of the master regulator CCR4−NOT. Given that blood lipid balance is one of the greatest risk factors for atherosclerosis, our result suggests a highly credible hypothesis for the mechanism linking *NTNG1* genetic variation to cerebral atherosclerosis progression that warrants validation through additional quantitative analysis and experimental tests. Discovering etiological mechanisms may in turn shed light on how cerebral atherosclerosis affects other brain phenotypes, such as dementia. More broadly, these results support the value of multi-omics studies for revealing risk factors and mechanisms for cerebral atherosclerosis and suggest that further genome-wide analyses in larger sample sizes and additional global populations are likely to discover additional genetic associations and pQTL loci.

## Figures and Tables

**Table 1:** Characteristics of ROS/MAP Dataset. Cerebral atherosclerosis was rated as none (0), mild (1), moderate (2), or severe (3). Education is the number of years of regular education at baseline. Post-mortem interval is time in hours between death to autopsy. Vascular risk factors is a composite measure of vascular risk comprising measures of hypertension, diabetes, and smoking history. Tangle density is average tangle density per mm^2^ over sampled cortical brain regions. Amyloid is average percent area occupied by amyloid *β* over sampled cortical brain regions.

Characteristic	N	Percent	
Sex			
Female Male	877 448	66.2 33.8	
Cognitive diagnosis at death * Normal cognition Mild cognitive impairment Alzheimer’s dementia Other dementia	397 297 522 23	30.0 22.4 39.4 1.7	
	Mean (SD)	Median	Range
Age at enrollment	80.4 (6.91)	80.8	63.0–102.2
Age at death	89.5 (6.56)	89.8	66.0–108.3
Education (years)	16.4 (3.60)	16.0	5.0–30.0
Post-mortem interval (hours)	9.1 (8.01)	6.6	0.0–98.3
Vascular risk factors	1.1 (0.84)	1.0	0.0–3.0
	N	Percent	
Gross infarct (Present)	585	44.2	
Microinfarct (Present)	499	37.7	
	Mean (SD)	Median	Range
Cerebral atherosclerosis	1.25 (0.81)	1.00	0.0–3.0
Alzheimer’s disease pathology			
Amyloid	4.1 (4.07)	3.1	0.0–22.9
Tangles	7.3 (8.79)	4.3	0.0–78.5

For quantitative variables, the mean, standard deviation (SD), median, and range are presented. For gross infarct and microinfarct, the number of participants with data (N) and the percent of participants with infarcts is presented. * Cognitive diagnosis was unavailable for 86 (6.49%) of the subjects.

**Table 2:** METAL meta-analysis. Estimated *β* coefficients from the independent ROS/MAP and Banner association analyses, and the result of inverse variance weighted meta-analysis of the two datasets using METAL.

	ROS/MAP		Banner		METAL	
SNP	*β* (SE)	*p*	*β* (SE)	*p*	Overall Effect (SE)	*p*
rs7531573	−0.20 (0.04)	3.89 × 10^−8^	−0.15 (0.13)	0.25	−0.19 (0.03)	1.93 × 10^−8^
rs12035149	−0.21 (0.04)	1.64 × 10^−8^	−0.12 (0.13)	0.38	−0.20 (0.04)	1.22 × 10^−8^
rs10881463	−0.20 (0.04)	3.40 × 10^−8^	−0.14 (0.13)	0.28	−0.20 (0.03)	1.84 × 10^−8^
rs11185092	−0.22 (0.04)	2.62 × 10^−9^	−0.12 (0.13)	0.38	−0.21 (0.04)	2.04 × 10^−9^
rs11185093	−0.22 (0.04)	3.26 × 10^−9^	−0.12 (0.13)	0.35	−0.21 (0.04)	2.35 × 10^−9^
rs12742040	−0.28 (0.04)	3.30 × 10^−10^	−0.14 (0.16)	0.36	−0.27 (0.04)	2.56 × 10^−10^
rs4274093	−0.27 (0.04)	7.39 × 10^−10^	−0.14 (0.16)	0.36	−0.26 (0.04)	5.51 × 10^−10^
rs6664221	−0.27 (0.04)	1.29 × 10^−10^	−0.13 (0.15)	0.38	−0.26 (0.04)	1.17 × 10^−10^

## Data Availability

Results of this study are available at https://synapse.org upon publication, accessed on 16 March 2020. Mediation R package: https://CRAN.R-project.org/package=mediation, accessed on 16 March 2020. MatrixEQTL R package: https://CRAN.R-project.org/package=MatrixEQTL, accessed on 16 March 2020. ROS/MAP phenotype data are available at https://www.radc.rush.edu, accessed on 16 March 2020. ROS/MAP genetic data are available at https://www.synapse.org/#!Synapse:syn17008936, accessed on 16 March 2020. ROS/MAP transcriptomic data are available at https://www.synapse.org/#!Synapse:syn8456704, accessed on 16 March 2020. ROS/MAP proteomic data are available at https://www.synapse.org/#!Synapse:syn17015098, accessed on 16 March 2020. Genotyping on participants from ADRCs are available at https://www.niagads.org/home, accessed on 16 March 2020.

## Appendix A

**Table A1:** ADGC independent analysis and meta-analysis. Estimated *β* coefficients from the independent ADGC association analysis, and the result of inverse variance weighted meta-analysis of the ROS/MAP, Banner, and ADGC datasets using METAL.

ADGC			METAL (ROS/MAP, Banner, ADGC)	
SNP	*β* (SE)	*p*	Overall Effect (SE)	*p*
rs7531573	0.02 (0.03)	0.65	−0.08 (0.02)	3.9 × 10^−4^
rs12035149	0.02 (0.03)	0.51	−0.92 (0.02)	5.4 × 10^−4^
rs10881463	0.02 (0.03)	0.61	−0.08 (0.02)	4.6 × 10^−4^
rs11185092	0.02 (0.03)	0.54	−0.91 (0.02)	2.2 × 10^−4^
rs11185093	0.02 (0.03)	0.55	−0.09 (0.02)	2.2 × 10^−4^
rs12742040	0.07 (0.04)	0.11	−0.09 (0.03)	1.3 × 10^−3^
rs4274093	0.06 (0.04)	0.18	−0.10 (0.03)	8.1 × 10^−4^
rs6664221	0.06 (0.04)	0.15	−0.90 (0.03)	4.5 × 10^−4^

**Table A2:** Summary of demographic and neuropathologic characteristics per cohort. Values are presented for the ROS/MAP, Banner, and ADGC samples (altogether and split by genotyping wave, or batch) included in the current analysis. Cerebral atherosclerosis semiquantitive scores range from 0 (none) to 3 (severe). Braak stage ranges from 0 to 6.

		Banner	ROS/MAP	ADGC (All Waves)	ADGC Wave 1	ADGC Wave 2	ADGC Wave 3	ADGC Wave 4	ADGC Wave 5	ADGC Wave 6	ADGC Wave 7
Sample Count		154	1325	1914	146	277	308	297	344	338	204
Age (years)	median	86	90	85	85	85	86	85	86	84	84
	mean	85.5	89.5	83.7	84.8	84.8	84.4	83.4	84.5	81.4	83.3
	SD	7.11	6.56	9.73	8.75	7.05	10.41	10.05	9.20	12.17	7.42
	range	66–103	66–108	47–111	57–105	65–98	55–111	47–103	56–102	52–109	67–102
Sex	N female	66	877	916	72	135	141	133	175	169	91
	N male	88	448	998	74	142	167	164	169	169	113
	% female	43%	66%	48%	49%	49%	46%	45%	51%	50%	45%
	% male	57%	34%	52%	51%	51%	54%	55%	49%	50%	55%
Cerebral Atherosclerosis	median	2	1	1	1	2	2	1	1	1	1
	mean	1.87	1.25	1.41	1.38	1.66	1.47	1.31	1.38	1.31	1.33
	SD	0.96	0.81	0.92	0.91	0.90	0.87	0.93	0.89	0.90	1.02
Cognitive Diagnosis at Death	% Normal	40%	30%	16%	38%	5%	14%	13%	21%	12%	16%
	% MCI	16%	22%	11%	12%	3%	7%	16%	13%	15%	5%
	% AD	44%	39%	47%	33%	72%	59%	44%	36%	46%	32%
	% Other Dementia	0%	2%	26%	18%	20%	20%	26%	29%	26%	47%
PMI (hours)	median	3.0	6.6	9.0	8.3	7.0	9.2	10.6	9.7	10.0	7.9
	mean	3.0	9.1	13.5	11.7	11.8	13.6	14.2	14.6	14.3	12.2
	SD	0.8	8.0	13.8	9.2	12.7	13.1	13.5	14.9	14.0	14.5
	range	1.5–5.5	0.0–98.3	0.0–99.0	0.5–37.5	1.3–81.8	0.0–63.7	0.0–72.0	1.3–96.0	0.0–96.0	0.0–99.0
	N missing	0	0	1112	107	194	220	205	210	133	43
Gross Infarct *	% present	44%	44%	20%	25%	22%	18%	20%	20%	19%	16%
	N missing	0	0	4	1	0	1	0	1	1	0
Microinfarct *	% present	−	38%	24%	27%	22%	23%	19%	22%	28%	27%
	N missing	−	0	3	1	0	1	1	0	0	0
Braak Stage	median	4	4	5	4	5	5	5	4	5	5
	mean	4.0	3.6	4.4	3.8	4.8	4.5	4.5	3.8	4.6	4.6
	SD	1.3	1.2	1.7	1.7	1.5	1.7	1.6	1.8	1.6	1.7
	N missing	0	0	3	0	1	0	1	1	0	0
CERAD Neuritic Plaque Density	none	21%	−	17%	27%	10%	16%	15%	20%	16%	17%
	sparse	8%	−	13%	12%	8%	12%	13%	22%	13%	11%
	moderate	18%	−	22%	20%	16%	23%	26%	26%	19%	21%
	frequent	52%	−	48%	41%	67%	49%	46%	32%	53%	51%
	N missing	3	−	1	0	0	0	0	1	0	0
CERAD confidence in AD	No AD	24%	23%	−	−	−	−	−	−	−	−
	Possible AD	25%	9%	−	−	−	−	−	−	−	−
	Probable AD	5%	35%	−	−	−	−	−	−	−	−
	Definite AD	40%	33%	−	−	−	−	−	−	−	−
	N missing	10	0	−	−	−	−	−	−	−	−

* The Banner infarct variable includes all infarct types.

**Table A3:** Formal comparison of cohort characteristics. Differences between ROS/MAP and the two other cohorts were tested using Pearson’s chi-squared test (categorical variables) or Welch *t*-test (quantitative variables).

	ROS/MAP v. ADGC					ROS/MAP v. Banner				
	χ^2^	*t*	df	N	*p*	χ^2^	*t*	df	N	*p*
% Female	105.7		1	3239	8.5 × 10^−25^	31.5		1	1479	2.0 × 10^−8^
Cognitive Diagnosis *	451.5		3	3153	1.5 × 10^−97^	10.0		3	1393	1.9 × 10^−2^
% Gross Infarct *	222.0		1	3235	3.4 × 10^−50^	0.0		1	1479	9.5 × 10^−1^
% Microinfarct *	71.9		1	3236	2.3 × 10^−17^	1.8		1	1479	1.9 × 10^−1^
Age		20.0	3196.9	3239	1.0 × 10^−83^		6.6	185.6	1479	3.2 × 10^−10^
Cerebral Atherosclerosis		−5.2	3048.0	3239	2.6 × 10^−7^		−7.7	179.3	1479	8.7 × 10^−13^
Braak Stage *		−14.9	3220.7	3229	2.6 × 10^−48^		−3.0	187.4	1479	2.9 × 10^−3^

* *p* < 0.001 in ROS/MAP—ADGC comparison and *p* > 0.001 in ROS/MAP—Banner comparison.
